# The Potential Correlation between Bacterial Diversity and the Characteristic Volatile Flavor Compounds of Sichuan Sauce-Flavored Sausage

**DOI:** 10.3390/foods13152350

**Published:** 2024-07-25

**Authors:** Lili Ji, Yanan Zhou, Qing Nie, Yi Luo, Rui Yang, Jun Kang, Yinfeng Zhao, Mengzhao Zeng, Yinhua Jia, Shirong Dong, Ling Gan, Jiamin Zhang

**Affiliations:** 1 Meat Processing Key Laboratory of Sichuan Province, College of Food and Biological Engineering, Chengdu University, Chengdu 610106, China; jilili@cdu.edu.cn (L.J.); zyn6089@163.com (Y.Z.); 13281811538@163.com (Q.N.); 18402833439@163.com (Y.L.); 13649049084@163.com (R.Y.); zhao_yinfeng@163.com (Y.Z.); 2 Key Laboratory of Natural Products and Functional Food Development Research, Sichuan Vocational College of Chemical Industry, Chengdu 646000, China; 13089063092@163.com; 3 Sichuan Stega Biotechnology Co., Ltd., Chengdu 610199, China; zengmengzhao@126.com; 4 Sichuan Fansaoguang Food Group Co., Ltd., Chengdu 611732, China; 15102854563@163.com (Y.J.); 15228991017@163.com (S.D.); 5 College of Veterinary Medicine, Southwest University, Chongqing 400715, China; gl9089@swu.edu.cn

**Keywords:** Sichuan sauce-flavored sausage, bacterial diversity, volatile flavor, core bacteria, high-throughput sequencing

## Abstract

The distinctive taste of Sichuan sauce-flavored sausage comes from an intricate microbial metabolism. The correlation between microbial composition and distinct flavor components has not been researched. The study used headspace solid-phase microextraction action with gas chromatography mass spectrometry to find flavor components and high-throughput sequencing of 16S rRNA to look at the diversity and succession of microbial communities. The correlation network model forecasted the connection between essential bacteria and the development of flavors. The study revealed that the primary flavor compounds in Sichuan sauce-flavored sausages were alcohols, aldehydes, and esters. The closely related microbes were *Leuconostoc*, *Pseudomonas*, *Psychrobacter*, *Flavobacterium*, and *Algoriella*. The microbes aided in the production of various flavor compounds, such as 1-octen-3-ol, benzeneacetaldehyde, hexanal, (R,R)-2,3-butanediol, and ethyl caprylate. This work has enhanced our comprehension of the diverse functions that bacteria serve in flavor development during the fermentation of Sichuan sauce-flavored sausage.

## 1. Introduction

As a ‘Chinese New Year commodity’ sausages sell over 6 million tons annually in China, especially the traditional air-dried sausages of Sichuan after December [1]. Sauce-flavored sausage belongs to a type of Sichuan-style sausage consumed in southwestern China. The source of sauce flavor mainly relies on local fermented condiments, such as Chinese yellow rice wine [2], Pixian broad bean paste [3], and Sufu [4]. During the processing and storage of sausages, beneficial microorganisms like lactic acid bacteria, *Staphylococcus* spp., *Micrococcus*, yeasts, and molds in these seasonings grow and metabolize, engaging in a series of biochemical reactions with proteins, fats, and carbohydrates, imparting excellent sauce flavor to the sausages [5].

During the fermentation process of sausages, microorganisms play a crucial role by influencing the physicochemical properties, amino acids, and volatile flavor compounds [6]. The core microorganisms of Chinese fermented sausages, such as lactic acid bacteria, *Staphylococcus* spp., and other microorganisms, are crucial for the unique flavor of sausages [7]. For example, *Staphylococcus putrefaciens*, *Staphylococcus carnosus*, and *Staphylococcus xylosus* can affect the formation of nitrites by producing nitrate reductase and catalase, thus influencing the color formation [8]. *Lactobacillus plantarum* and *Lactobacillus casei* can significantly degrade myofibrils and sarcoplasmic proteins, promote the production of free amino acids, and then metabolize them into aldehydes, alcohols, and acids, ultimately increasing the content and types of flavor compounds [9]. In addition, sausages also have distinct regional characteristics and flavors, which may be due to differences in microbial communities during processing. The dominant sausage communities in the Hunan region are mainly *Staphylococcus* spp., *Lactobacillus*, *Solanum torvum*, and *Brochothrix* [10]. The dominant bacteria in the northeast region are mainly *Lactobacillus* spp., *Staphylococcus* spp., *Leuconostoc* spp., *Lactococcus* spp., and *Weissella* spp. [11]. However, there is limited research on the dynamic evolution of microorganisms and flavor compounds in Sichuan-style naturally fermented sausages. Exploring the core microbial community and key flavors of sauce-flavored sausages can help to better understand their complex fermentation process.

**Figure 1:**
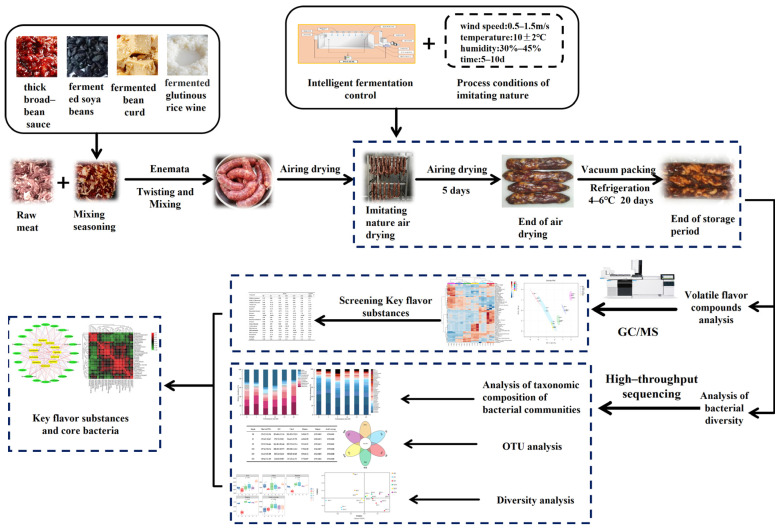
Sauce-flavored sausage processing technology.

In this study, we aim to investigate the relationship between changes in microbial communities and the generation of volatile flavor compounds at different fermentation times. The changes in volatile flavor compounds and microbial communities were analyzed using headspace solid-phase microextraction action with gas chromatography mass spectrometry (HS–SPME–GC–MS) and high-throughput sequencing (HTS), respectively. Meanwhile, the correlation between bacterial communities and characteristic flavor compounds was intuitively expressed based on the Cytoscape network diagram. Figure 1 shows the navigational map of this study. This study provides data support for the processing technology of traditional fermented meat products in China and lays the foundation for the quality and safety of sausage products.

## 2. Materials and Methods

### 2.1. Materials and Reagents

#### 2.1.1. Experiment Materials

Sausage ingredients: The foreleg meat and back fat of captive feed pigs aged 8 months were purchased from Sichuan Gaojin Food Co., Ltd. (Suining, China) and transported to the laboratory under refrigerated conditions at 4 °C.

Seasonings: The fermented black beans were purchased from Chengdu Taihefang Brewing Co., Ltd. (Chengdu, China); the doubanjiang was purchased from Sichuan Dandan Pixian Douban Group Co., Ltd. (Chengdu, China); the fermented glutinous rice and fermented bean curd were both purchased from Chengdu Guoniang Food Co., Ltd. (Chengdu, China); the thirteen-spice blend and salt were obtained from local markets in Chengdu, Sichuan Province, China.

#### 2.1.2. Experiment Reagents

Chemical reagents: thiobarbituric acid (TBA), disodium ethylene diamine tetra-acetic acid (disodium EDTA), hydrochloric acid, trichloroacetic acid, absolute ethyl alcohol, sodium thiosulfate, and other reagents were purchased from Chengdu chron Chemicals Co., Ltd. (Chengdu, Sichuan, China); potassium hydrogen phthalate (pH = 6.86 buffer) and mixed phosphate (pH = 4 buffer) were purchased from Inesa Scientific Instrument Co., Ltd. (Shanghai, China); 2,4,6-Trimethylpyridine, phenol, and sodium citrate were purchased from Sigma-Aldrich Shanghai Co., Ltd. (Shanghai, China).

Microbial culture media: Plate count agar, MRS agar, mannitol fermentation agar, and agar were all purchased from Beijing Aoboxing Bio-Tech Co., Ltd. (Beijing, China).

### 2.2. Processing of Sauce-Flavored Sausage and Sampling

The fresh pork foreleg meat and back fat were cut into lean meat strips of 4 mm× 4 mm × 20 mm and fat cubes of 4 mm × 4 mm× 4 mm, respectively. Secondly, the sauces for the sausages were created in the laboratory using a blend of soy bean paste, fermented bean curd, tempeh, mash, and thirteen spices, which provide a rich fermented flavor. The sausages were prepared using shredded and diced pork (4:1), 2.5% salt, and 10% seasoning sauce from Sichuan Jiuda Salt Co., Ltd. (Chengdu, China). The minced meat mixture was then stuffed into fresh pig intestines using an automatic enema machine. The sausages were portioned into small knots using cotton strings, resulting in a final weight, length, and diameter of approximately 0.15 kg, 15 cm, and 2 cm per knot. The sausages were placed in a fully automated air-drying fermentation room (BFJX-500; Hangzhou Aibo Mechanical Engineering Co., Ltd. Hangzhou, Zhejiang, China) at a temperature of 10 ± 2 °C and a relative humidity of 30–45%. Samples were taken after one day. After being removed for 5 days, the items were vacuum-sealed and stored in an environment with a temperature of 15 ± 3 °C and a relative humidity of 35–40% to protect them from light. Samples were taken at 10, 15, and 25 days, frozen, and stored at −80 °C for future use.

### 2.3. Evaluation of Physicochemical Characteristics

#### 2.3.1. The Determination of the pH

The pH of the sausage was measured using a direct-insertion pH meter (Testo 205 pH meter, German Instrument Co., Ltd., Shenzhen, China) [12]. The pH meter was calibrated before use. The pH of the standard buffer used to calibrate the instrument was selected to be 4.00 and 6.86, respectively.

#### 2.3.2. The Determination of the Water Activity

The water activity of the sausage was measured using the method described by Ji et al. [13]. The sausage sample was thoroughly chopped into approximately 0.2 mm in size, and 2 g of each sample was evenly spread onto a water activity meter (HD-5, Wuxi Huake, Wuxi, China) for measurement. The experiment was repeated three times, and the average value was recorded.

#### 2.3.3. Color Measurement

The color characteristics of the sausage samples were measured using the Chroma Meter CR-400 (Konica Minolta Holdings, Inc., Tokyo, Japan). The light source used for equipment and instrument testing is D65, with an observer angle of 2° and a measurement diameter of 8 mm (about 0.5 mm^2^ area). The color parameters for evaluation include redness (a*), yellowness (b*), and lightness (L*). Before using the equipment, it was calibrated with a standard white plate (CR–A43), where the calibration values were Y = 86.6; x = 0.3196; y = 0.3378.

### 2.4. Determination of Protein Carbonyl Content and Thiobarbituric Acid Reactive Substances

The carbonyl group was measured using the method described by Zhang et al. [14] The amount of carbonyl in the sample was expressed as nmol/mg protein using an absorption coefficient of 22,000 M^−1^ cm^−1^ for protein hydrazones. The TBARS (thiobarbituric acid reactive substances) were measured according to the method described by Chen et al. [15] to assess the lipid oxidation level during sausage processing. The absorbance of the test sample solution was measured using an ultraviolet spectrophotometer (UV-1100, Mepheda Instrument Co., Ltd., Shanghai, China) at 532 nm. The TBARS were then calculated using the following formula:

(1) $$\mathrm{TBARS}\;(\mathrm{mg}/\mathrm{kg})=\;(A_{532}/\mathrm{Ws})\times 9.48$$

In Equation (1), A_532_ represents the absorbance of the test solution at 532 nm, Ws represents the weight of the sausage (g), and “9.48” is a constant derived from the dilution factor and the molar extinction coefficient (152,000 M^−1^ cm^−1^) of the red thiobarbituric acid reaction product.

### 2.5. Determination of Free Amino Acids

The detection of free amino acids was conducted based on the method described by Xiao et al. [16] with slight modifications. In total, 5 g of sausage was weighed into a 30 mL pressure-resistant glass tube with a screw lid. Next, 15 mL of a 6-molar hydrochloric acid solution was added, along with 3 drops of phenol, and thoroughly mixed. The glass tube was cooled in an ice bath for 5 min, emptied, then filled with nitrogen and sealed with the screw cap. The glass tubes were heated in an electric blast thermostat at 110 °C for 22 h, then cooled to ambient temperature, filtered through double quantitative filter paper, and diluted to 50 mL with ultrapure water. A total of 1 mL of the uniform liquid was transferred into a conical flask and dehydrated under decreased pressure using a rotary evaporator at 120 revolutions per minute and 45 °C. After complete evaporation, the substance was diluted in 1 mL of ultrapure water, dried under decreased pressure, and then evaporated until completely dry. The solid was dissolved by adding 1 mL of pH 2.2 sodium citrate buffer, drawn up with a sterile syringe, passed through a 0.22 µm sterile filter, and then moved to the instrument injection bottle for testing. The solution was examined using an amino acid analyzer (LC98-I, Beijing Wenfen Analytical Instrument Technology Development Co., Ltd., Beijing, China) with a UV-Vis detector and a C18 ion-exchange chromatographic column. The free amino acids were identified and measured based on the retention time and peak area of amino acid standards. The free amino acid concentration was reported as mg/100 g of sausage.

### 2.6. Analysis of Volatile Compounds

The detection of volatile compounds in sausages was carried out according to the method described by Zhao et al. [17], with slight modifications. HS–SPME–GC–MS (7890B GC, 5977A MS; Agilent Technologies, Santa Clara, CA, USA) is used to detect volatile chemicals in sausages. A total of 3.0 g of sausage was weighed into a 20 mL vial with headspace, 1 μL of 2 μg/μL 2,4,6-trimethylpyridine was added as an internal standard, then the small bottle was placed in a thermostat and equilibrated at 40 °C for 15 min. Headspace solid-phase microextraction was conducted using a DVB/CAR/PDMS extraction needle (2 cm, 50/30 μm, Supelco, St. Louis, MO, USA) at 60 °C for 30 min. The volatile substances in SPME fibers were drawn into the injector using the splitless mode of gas chromatography at 250 °C for 5 min, with helium as the carrier gas flowing at a rate of 1.0 mL/min. The volatile compounds were analyzed using GC-MS (30 m × 0.25 mm × 0.25 μm; Agilent, Santa Clara, CA, USA) with an HP–5MS column. The column temperature started at 35 °C and was held for 2 min, then increased to 80 °C at a rate of 2 °C per minute and kept for 3 min. Subsequently, the temperature was raised to 110 °C at a rate of 5 °C per minute and maintained for 32 min, followed by an increase to 180 °C at a rate of 12 °C per minute and held for 2 min. The temperature was ramped up to 220 °C at a rate of 10 °C/min and maintained for 1 min. The mass spectrometer was operated with an ion source temperature of 230 °C and a quadrupole temperature of 150 °C. It performed a mass scan from *m*/*z* 40 to 450 using an electron energy of 70 eV. Volatile compounds were identified based on retention indices (RI), authentic standards, and mass spectra matching the NIST 14.0 database (National Institute of Standards and Technology, Gaithersburg, MD, USA). The RI of each volatile compound was calculated relative to that of 2,4,6-trimethylpyridine (C8H11N, Agilent Technologies, Beijing, China). Peak area normalization was used to quantify the total ion flux chromatograms in order to determine the relative amount of each component. The concentration of each volatile compound was calculated as follows:

(2) $$\mathrm{Concentration}\;(\mathrm{mg}/\mathrm{kg})=\;\frac{P\times m1}{m2}\times 10^{3}$$

In Equation (2), *P* is the ratio of the peak of each volatile compound to the peak area of 2,4,6-trimethylpyridine, *m*1 is the added mass (μg) of the standard compound, and *m*2 is the weight of the sausage sample (g).

### 2.7. Calculation of ROAV Value

Relative odor activity value (ROAV) is a measure used in sensory analysis to quantify the intensity of odor or aroma compounds in relation to a reference substance [18]. The contribution of volatile substances to the overall flavor of a sample can usually be determined by calculating the ROAV value of the substance [19]. The ROAV of volatile flavor compounds that most significantly affect the overall flavor of the sample is set to 100. Compounds with an ROAV ≥ 1 are considered key volatile aroma compounds in the sample, while compounds with 0.1 < ROAV < 1 are considered to have a modifying effect on the flavor of the sample [20]. The ROAV was calculated according to the following formula:

(3) $$\mathrm{ROAV}=\;(C_{A}/T_{A})\times \;(T_{S}/C_{S})\times 100$$

In Equation (3), C_A_ represents the relative percentage content of compound A, T_A_ represents the odor threshold of compound A (mg/kg), and T_S_ and C_S_ are the aroma thresholds and relative percentage contents of the compounds that contribute the most to the sample, respectively.

### 2.8. Bacterial Communities

#### 2.8.1. Total Bacteria, Lactic Acid Bacteria, and *Staphylococcus* spp. Counts

The sausage samples were collected throughout the entire processing and subjected to consecutive dilutions (10^−1^ to 10^−6^), followed by cultivation on suitable microbial culture media. The methods described by Shao et al. [21] and Xiao et al. [22] for analyzing the total bacterial count, lactic acid bacterial count, and staphylococcus count in sausages were referenced, with slight modifications. Each sausage sample (10 g) was aseptically diluted 10-fold with 90 mL of sterile isotonic saline and homogenized in a SCIENTZ-09 sterile homogenizer (Ningbo Scientz Biotechnology Co., Ltd., Ningbo, China) for 5 min. Then, a continuous 10-fold sterile diluent was prepared and analyzed as follows: LAB counts on MRS agar incubated at 30 °C for 48 h; Staphylococcus spp. on mannitol salt agar incubated at 30 °C for 48 h; and total bacterial counts on plate count agar incubated at 37 °C for 48 h.

#### 2.8.2. DNA Extraction and Sequencing

After mincing, the sauced meat with a balanced ratio of fat to lean mass, was accurately weighed to 3 g in a sterile bag, and 27 mL of 0.85% NaCl solution was added to mix thoroughly. The homogenized solution was centrifuged at 4 °C, 12,000 g/min for 10 min. The supernatant was removed, the microbial precipitate was collected, and microbial DNA was extracted using the HiPure Fecal DNA Kit (d3141, Guangzhou Meiji Biotechnology Co., Ltd., Guangzhou, Guangdong, China). The quality of DNA was tested with a nano-microspectrophotometer (nanodrop 2000; Thermo Fisher, Waltham, MA, USA). The primers 341F (CCTACGGGGNGGCWGCAG) and 806R (GGACTACHVGGGTATCTAAT) were used to amplify the V3─V4 region of the bacterial 16S rRNA gene. PCR products were purified using AMPure XP beads, quantified using the ABI StepOnePlus Real-Time PCR System (Life Technologies, Carlsbad, CA, USA), and sequenced using the PE250 mode on the NovaSeq 6000. The 16S rRNA gene data were processed using Usearch software (version 11) to remove low-quality data. The OTU sequences were taxonomically classified using the naive Bayesian model of the RDP classifier (version 2.2) using the SILVA and UNITE databases.

### 2.9. Data Processing and Statistical Analysis

Three batches of sausage were prepared, and samples of sauce-flavored sausage at different fermentation stages were tested. The sausage samples tested separately for each fermentation period were bioreplicated 3 times. The data were analyzed for significance using one-way ANOVA and Tukey’s test for multiple comparisons in SPSS 26.0. Significance was set at *p* < 0.05. Data were expressed as the mean ± SD. Physicochemical characteristics were assessed with GraphPad Prism 9.0.0, amino acids were examined using Origin 2021, and microbial communities were evaluated with Kidio BioCloud (https://www.omicshare.com/tools/, accessed on 24 November 2022). MetaboAnalyst 5.0 was utilized for the analysis and visualization of volatile compounds.

## 3. Results and Discussion

### 3.1. Dynamics of Physicochemical Parameters

#### 3.1.1. The Dynamic Changes in pH, Water Activity, and Color

**Figure 2:**
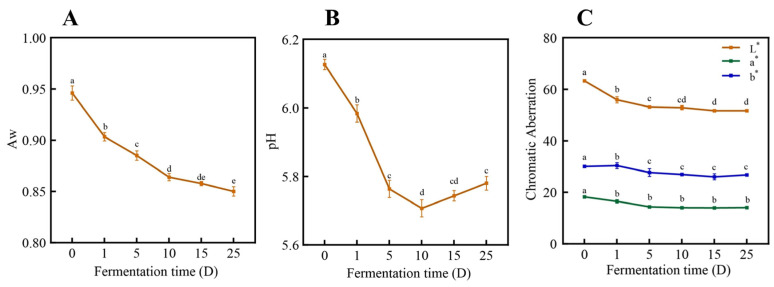
(**A**) Changes in the water activity of sausage at different stages; (**B**) changes in the pH of sausage at different stages; (**C**) changes in the color of sausage at different stages. Note: different lowercase letters indicate significant differences in the sausage at different time points.

With respect to the dynamic changes in pH, water activity, and color, as depicted in Figure 2A, the aw decreased from 0.949 to 0.846 in the sauce-flavored sausage over the entire fermentation period (*p* < 0.05). Similar findings were also observed by Hu et al. [23,24], and they believe that the decreases in aw resulted from water migration within the sausage and evaporation of surface water during fermentation. The pH exhibits a trend of initially decreasing, followed by an increase (Figure 2B). The pH sharply dropped from 5.79 to 5.54 on the 10th day, then increased to 5.59 on the 15th day and remained constant thereafter. The decrease in the pH at the initial and middle stages of fermentation (0–10 d) was associated with the growth of the microorganism that produces mixtures of organic acids, and the increase in the pH during the later stage (10–25 d) could have been caused by the formation of nitrogenous compounds from protein degradation and the decomposition of organic acids [25]. The evolution of lightness, redness, and yellowness during the ripening and storage of sauce-flavored sausages is shown in Figure 2C. Consistent with previous studies by Yim et al. [26], the L* value and a* value of sausage decreased significantly after 0–10 days (*p* < 0.05), which may be ascribed to water loss during production. Additionally, during the processing, the gradual deepening of protein and lipid oxidation is also a major factor leading to the decrease in sausage color [11,27].

#### 3.1.2. Changes in TBARS and Protein Carbonyl Groups

**Figure 3:**
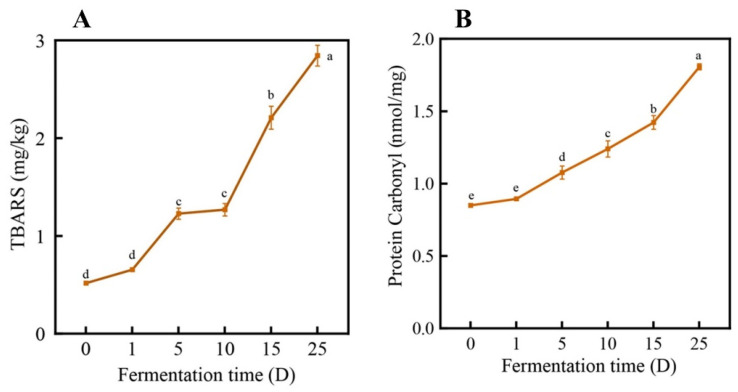
(**A**) The changes in TBARS of the sausage at different time periods; (**B**) the changes in the protein carbonyl content of the sausage at different time periods. Note: different lowercase letters indicate significant differences in the sausage at different time points.

TBARS content, which reflects the content of malonaldehyde, one of the degradation products of lipid hydroperoxides and peroxides formed during the oxidation of polyunsaturated fatty acids, is widely used as an indicator of the degree of lipid oxidation [28]. Figure 3A illustrates the TBARS values of the sausage at different fermentation periods. As the fermentation time extends, the TBARS values significantly increase (*p* < 0.05). The reason for this could be that during the sausage fermentation stage, fat is hydrolyzed by endogenous enzymes, leading to the production of a large amount of unsaturated fatty acids, which undergo oxidation, resulting in a rapid increase in TBARS values. The carbonyl content is a significant indicator of protein oxidation. The oxidative degradation of amino acids, particularly arginine, lysine, histidine, and proline, can produce carbonyl compounds that can impact the functioning of meat proteins [29]. From Figure 3B, it can be observed that throughout the entire fermentation and storage period, the carbonyl content gradually increases with prolonged time.

### 3.2. Analysis of Free Amino Acids

**Figure 4:**
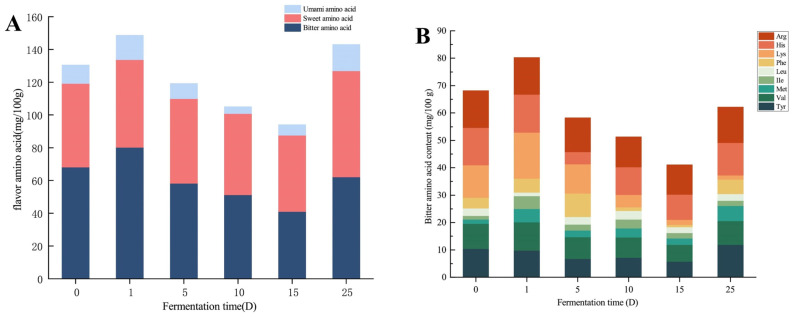

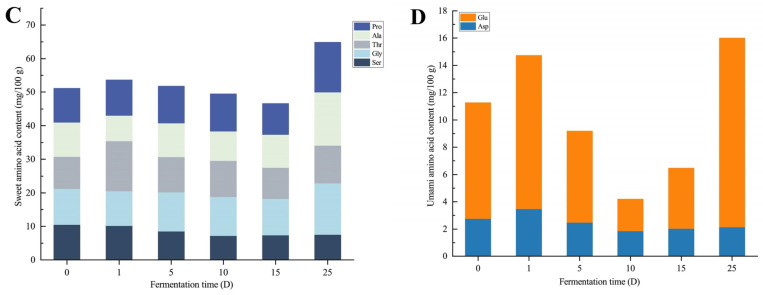
Changes in free amino acid content in different fermentation periods of sauce-flavored sausage. (**A**) The content of amino acids in taste changed; (**B**) the content of different kinds of bitter amino acids changed; (**C**) different kinds of sweet amino acid content changed; (**D**) The amino acid content of different kinds of umami flavor changed.

Amino acids play a crucial role as taste molecules in fermentation systems, as evidenced by various research studies [30]. Endogenous and exogenous enzymes aid in the breakdown of proteins in meat during fermentation. Peptides, amino acids, and tiny molecules play a role in creating taste compounds [31,32]. This study analyzed the levels of 16 different free amino acids, such as aspartic acid (Asp), glutamic acid (Glu), serine (Ser), glycine (Gly), histidine (His,), arginine (Arg), threonine (Thr), alanine (Ala), proline (Pro), tyrosine (Tyr), valine (Val), methionine (Met), ileucine (Ile), leucine (Leu), phenylalanine (Phe), and lysine (Lys). Figure 4A shows a notable increase in total amino acids on day 1, followed by a drop and a return to the initial level by day 25. The increase in total amino acids could be attributed to the high growth of *Lactobacillus* microbes in the initial phase. This rapid increase results in the generation of lactic acid, causing a decrease in the pH of the sausage. Consequently, it hinders the function of bacterial aminopeptidases and decreases the liberation of amino acids in the fermentation process. The subsequent rise could be due to the microbial decomposition of metabolized proteins, peptides, and amino acids, leading to an increase in their concentration [33].

According to their taste characteristics, FAAs can be mainly classified into umami FAAs (Asp and Glu), sweet FAAs (Ser, Thr Ala, Lys, Pro, and Gly), bitter FAAs (BFAAs; Phe, His, Arg, Val, Met, Tyr, Ile, and Leu), and the odorless amino acid (Cys) [34]. Figure 4A shows that the changes in the contents of bitter amino acids, sweet amino acids, umami amino acids, and total free amino acids are consistent. During fermentation, the content of sweet amino acids and bitter amino acids increases, which is consistent with the results of An et al. [35]. After day 25, the total amount of sweet and amino acids was greater than that of bitter amino acids. As for bitter amino acids, it can be seen in Figure 4B that the content of bitter amino acids was the highest on the first day and the lowest on the fifteenth day. After processing, the contents of Arg, His, Tyr, and Val were higher. Sweet amino acids (Pro, Ala, Gly) showed higher content on day 25 (Figure 4C); the content of umami amino acids is at its lowest on the 10th day and peaks on the 25th day, with Glu being the predominant component. This could be attributed to the rich Glu content in the seasoning (sufu) of the sausage, leading to an increase in Glu content in the sausage (Figure 4D) [36]. In addition, a total of seven essential amino acids were detected in the sauce-flavored sausage. The content of Ile decreased over time, while the levels of Val, Leu, Lys, Met, Phe, and Thr increased.

### 3.3. Analysis of Volatile Flavor Compounds

**Figure 5:**
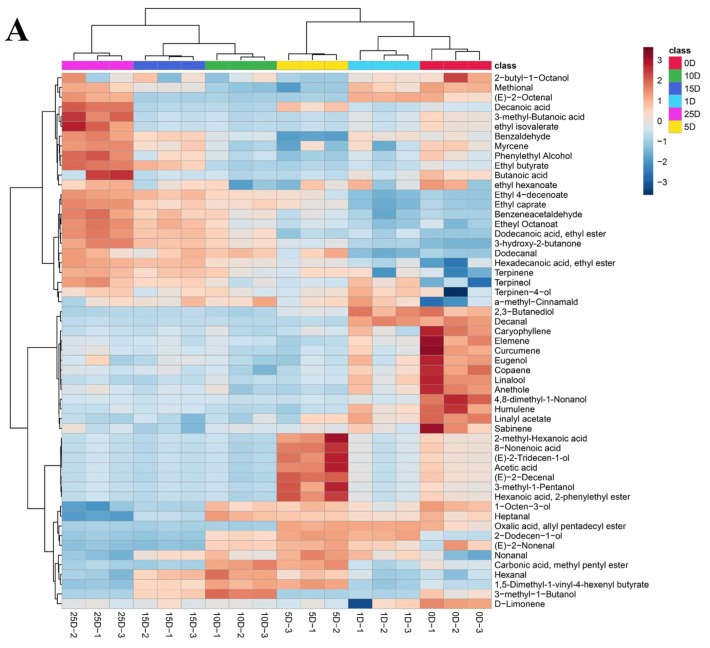

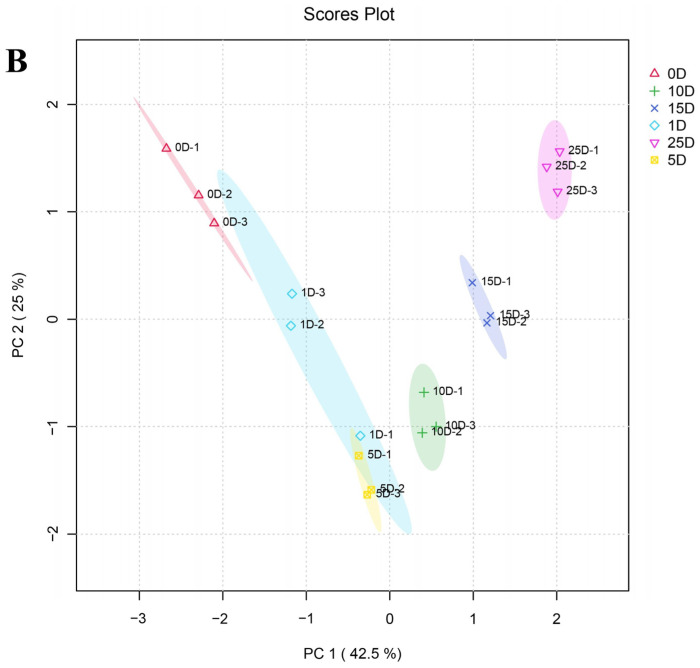
Differences in volatile compounds between fermentation periods. (**A**) Figure shows the analytical heat map of volatile compounds; the horizontal axis represents the different samples studied, the letters on the vertical axis represent the volatile flavor compounds, and the color scale indicates the nature of the correlation, with 4.00 indicating the maximum relative content of flavor (red) and −4.00 indicating the minimum relative content of flavor (blue); (**B**) principal component cluster analysis plot of volatile compounds for different fermentation periods.

HS–SPME–GC–MS analysis was used to detect the volatile flavor components produced during sausage preparation. Figure 5A shows the detection of 55 chemicals in sausage fermentation. These include 12 aldehydes, 12 alcohols, 13 esters, six acids, one ketone, and 11 other chemicals. Alcohols, aldehydes, esters, acids, and ketones are identified as the primary compounds that influence the taste of traditional Chinese air-dried sausages [10]. Furthermore, olefins are also present in addition to these volatiles. These chemicals are typically formed by the degradation of substrates such as proteins, carbohydrates, and macromolecules, as well as through lipid oxidation, microbial esterification, and the addition of flavorings [37].

Aldehydes are produced by the oxidation of unsaturated fatty acids and the breakdown of amino acids. They have a low odor threshold and play a key role in the flavor of sausages [38]. Throughout the fermentation process, the main components include benzaldehyde, methional, benzeneacetaldehyde, hexanal, and nonanal. Both benzaldehyde and benzeneacetaldehyde are produced from the degradation of phenylalanine and are believed to enhance the flavor of the sausage. Benzaldehyde manifests a delightful almond flavor, while benzeneacetaldehyde showcases floral, rose, and honey aromas [39,40]. During the late stage of fermentation, benzaldehyde and benzeneacetaldehyde exhibit higher levels. Furthermore, five kinds of fatty aldehydes, especially some short-chain fatty aldehydes (C5–C9) derived from lipid oxidation with low odor threshold values, might be vital to the sausage flavor [41]. Among them, hexanal has the odor of beef fat, grass, and fat, while nonanal has a lower threshold and is an oxidation product of oleic acid, making a significant contribution to the odor of sausages [42].

The unsaturated alcohols in alcohols typically have a low perception threshold and significantly impact the product’s flavor [33]. The primary alcohols identified during the sausage-making process were 2,3-butanediol, linalool, phenylethanol, 4-pinitol, and 2-butyl-1-octanol. On the 25th day, the phenylethanol content was predominant in the sausage, imparting a floral aroma with a relatively low odor threshold, which influenced the overall flavor. Other alcohols have higher thresholds and typically do not impact the overall flavor of the sausage.

For esters, most of the detected compounds were ethyl esters, which are commonly associated with adding fruity flavors and masking rancid odors [43,44]. They can be synthesized either by the esterification of alcohols with fatty acids or through alcohol acetyltransferase using acetyl-CoA and higher alcohols as substrates during fermentation [41]. Throughout the fermentation process, ethyl butyrate, ethyl octanoate, ethyl hexanoate, and ethyl caprate are all notably abundant. Short-chain fatty acids (C < 6) have greater implications in flavor development due to the very strong odors and to their lower threshold values [45]. In this study, 3-methyl-butyric acid, acetic acid, and butanoic acid have a significant impact on the flavor of the sausage. One ketone, specifically 3-hydroxy-2-butanone, was found. It is produced through the metabolism of citrate and lactose in the presence of LAB or through amino acid catabolism [46]. The concentration of 3-hydroxy-2-butanone rises as fermentation time advances. Moreover, numerous spices include unique volatile flavor molecules, such as d-limonene, copaene, caryophyllene, and anethole. The chemicals enhance the sausage’s flavor by adding a nice scent and sweetness [47].

PLS stands for partial least squares analysis, DA refers to discriminant analysis, and PLS-DA is a supervised method that combines both techniques to address classification and discriminant issues. It is commonly utilized in food research [48]. Unsupervised principal component analysis was used to cluster and downscale volatile compounds in sausages from various fermentation periods. Figure 5B shows that PC1 and PC2 accounted for 42.5% and 25% of the variance, respectively, totaling 67.5% of the total variance. During the fermentation process, the volatile compounds of the sausages were clearly differentiated, with significant differences in flavor profiles at different times. This shows that the flavors got better over time. The 25 D samples had more volatile compounds than the samples from the other fermentation periods.

### 3.4. Screening Key Flavor Substances

The study found that compounds with a relative odor activity value (ROAV) ≥ 1 are an important contributor to the perceived flavor. ROAV was used to identify the key flavor substances in Sichuan sauce-flavored sausage, according to the references. Table 1 lists the 20 key volatile flavor compounds with an ROAV ≥ 1 in Sichuan-flavored sausages at different periods, including 3-ethyl-2-pentanol, (R,R)-2,3-butanediol, 3-methyl-2-hexanol, 1-octen-3-ol, eucalyptol, linalool, phenylethyl alcohol, hexanal, methional, octanal, benzeneacetaldehyde, nonanal, trans-2-decenal, 2,4-decadienal, ethyl butyrate, ethyl hexanoate, ethyl caprylate, d-limonene, estragole, and anethole. The characteristic flavor compounds in sausages are primarily alcohols, aldehydes, and esters. Among them, eight are alcohols, indicating that the flavor of Sichuan-flavored sausages is mainly influenced by alcohols. In the sausage samples, the average ROAV values of the alcohol compounds, from highest to lowest, are eucalyptol, octanal, 1-octen-3-ol, linalool, phenylethyl alcohol, 3-methyl-2-hexanol, and 3-ethyl-2-pentanol. After the fermentation period (25 days), the alcohols with significant contributions to the sausage flavor were Linalool and 1-octen-3-ol. Linalool comes from the added spices, while 1-octen-3-ol is an oxidized product of linoleic acid and mainly provides flavors of mushroom, rose, and hay [49]. For the aldehyde compounds in sausages, their average ROAV values from high to low are methional, 2,4-decadienal, hexanal, nonanal, benzeneacetaldehyde, and trans-2-decenal. Among the key volatile compounds in sausages, methional contributes the most to the flavor, with a cooked potato aroma and meaty flavor, possibly formed by the metabolism of amino acids by Lactococcus lactis [50,51]. Benzeneacetaldehyde is a branched aldehyde that provides a grilled meat flavor to sausages [49]. Hexanal is derived from the degradation of linoleic acid, offering a fresh grassy taste. Nonanal, originating from the beta-oxidation of oleic acid, provides grassy, fatty, and buttery notes to the sausage [52]. Esters typically have characteristic fruity and sweet flavors, mainly formed by the esterification of acids and alcohols in meat products [53]. During the sausage processing, the esters with higher contribution values from highest to lowest are ethyl caprylate, ethyl hexanoate, and ethyl butyrate. Additionally, other compounds such as d-limonene, estragole, and anethole, which may come from spices, have high ROAV values, and contribute to the flavor formation of sausages. In summary, during different periods, various key flavor compounds in sauce-flavored sausages contribute to the overall flavor formation. This diversity is likely primarily due to differences in environmental factors such as temperature and oxygen levels during the processing. Therefore, it is necessary to further explore the impact of environmental factors on the flavor formation of Sichuan-style sauce-flavored sausages.

### 3.5. Bacterial Communities

#### 3.5.1. Total Bacteria, Lactic Acid Bacteria, and *Staphylococcus* spp. Counts

**Figure 6:**
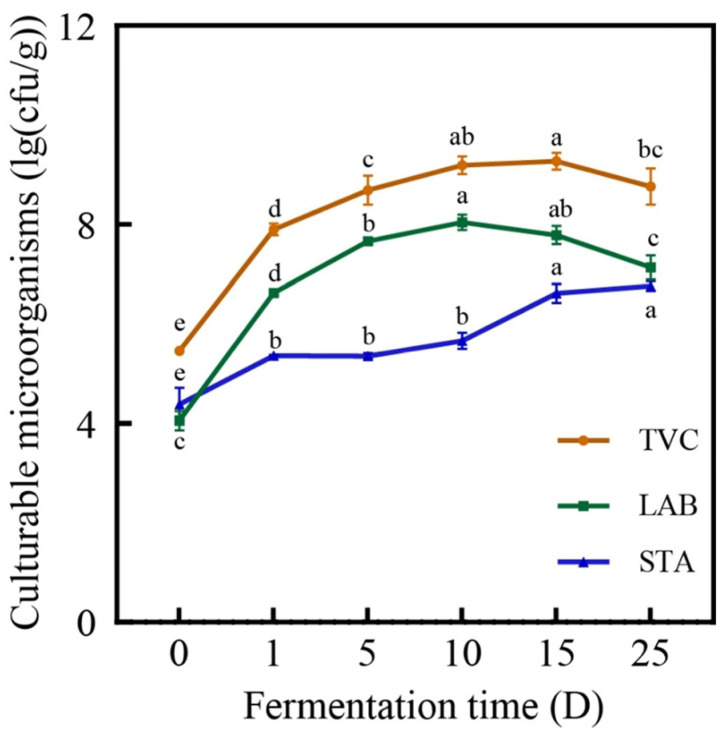
The changes in the total bacterial count, lactic acid bacteria count, and *Staphylococcus* spp. count of the sausage at different time periods. Note: different lowercase letters indicate significant differences in the sausage at different time points.

There are literature reports that in the early stage of sausage production, relatively low pH can effectively inhibit the growth and reproduction of harmful microorganisms, such as biogenic amines and nitrosamines [54]. The pH changes mentioned earlier are exactly opposite to the changes in the number of microorganisms shown in Figure 6. In the early stage of fermentation, microorganisms (such as lactic acid bacteria and *Staphylococcus* spp.) rapidly multiply and metabolize carbohydrates to produce organic acids, such as lactic acid, which rapidly lowers the pH of sausages. This is consistent with the research of Sun et al. [55]. As shown in the Figure 6, the countable colony numbers of the sausage exhibit a trend of initially increasing and then decreasing during the processing. From day 0 to day 15, the counts significantly rise (*p* < 0.05), increasing from 5.460 (Log CFU/g) at D0 to 9.273 (Log CFU/g) at D15. However, by the end of processing, the counts decrease to 8.763 (Log CFU/g) at D25. This decline in later storage stages could be due to the continuous reduction in moisture content, which inhibits microbial growth. *Lactobacillus* and *Staphylococcus* are significant genera in Sichuan sausages for creating distinct flavors [17]. For lactic acid bacteria, there is a significant increase (*p* < 0.05) from day 0 to day 10, rising from 4.053 (Log CFU/g) to 8.039 (Log CFU/g). However, by the end of the later storage period (D25), the count decreases to 7.133 (Log CFU/g). Lactic acid bacteria are ideal microorganisms in fermented sausages that can lower the pH or produce antibacterial compounds to inhibit the growth of undesirable microorganisms [56]. The growth pattern of *Staphylococcus* spp. is similar to that of lactic acid bacteria, showing a gradual increase over time, rising from 4.377 (Log CFU/g) at D0 to 6.764 (Log CFU/g) at D25. It is noteworthy that the number of lactic acid bacteria is higher than that of *Staphylococcus* spp., which is consistent with the findings of Shao et al. [11]. This may be due to the production of lactic acid by lactic acid bacteria during the growth process, which lowers the pH of sausages and inhibits the growth of some *Staphylococcus* spp. [12].

#### 3.5.2. Analysis of Microbial Diversity

**Figure 7:**
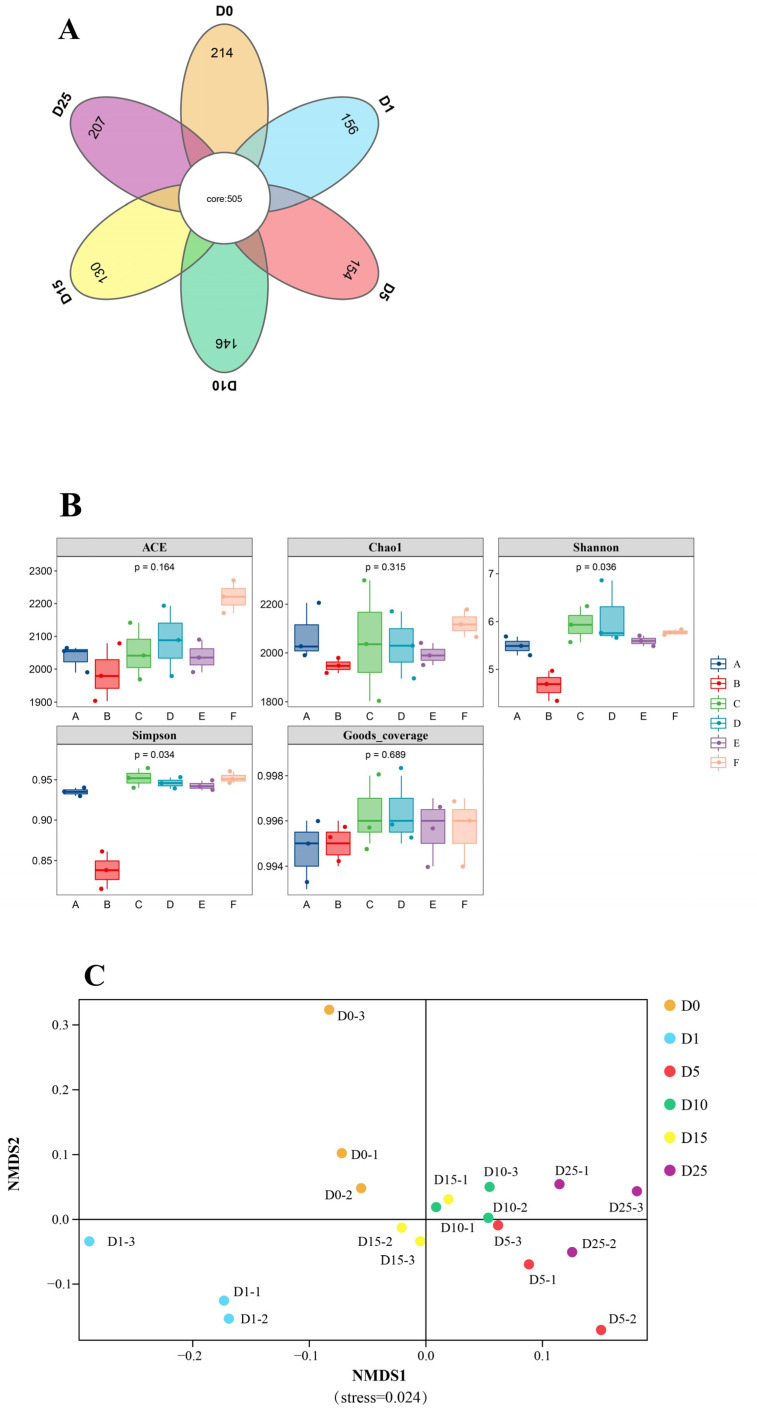
These figures show the differences in microbial diversity among six samples. (**A**) Venn diagram of the sample; (**B**) box-plot analysis of alpha diversity, where A, B, C, D, E, and F represent samples D0, D1, D5, D10, D15, and D25, respectively; (**C**) a plot of non-metric multidimensional scaling analysis of beta diversity.

Bacterial diversity and community structure were analyzed using MiSeq sequencing during the processing of six sausages. The Venn diagram in Figure 7A shows a total of 505 operational taxonomic units (OTUs) across all samples, indicating high bacterial diversity. The number of unique OTUs varied for each cycle. The number of unique OTU per cycle from D0 to D25 were 214, 156, 154, 146,130, and 207, respectively. Table 2 indicates that the OTU coverage for all six samples exceeds 99%, suggesting that all bacteria present in the samples are detectable. This sequencing result accurately reflects the presence of bacteria in the samples. Alpha diversity quantifies the variety and abundance of species in specific samples. The Chao1 and ACE indices are indicators of species richness, where higher values suggest increased diversity. The Shannon and Simpson indices together represent species richness and evenness, where higher values suggest greater species diversity in the samples. The Chao1 index and ACE index were highest on day 25, suggesting that the species richness of this sample was greater compared to the preceding periods, as indicated in Table 2. The Shannon index was higher on day 5, while the Simpson index was higher on day 25. The data were analyzed using box-and-whisker plots, which visually verified the coherence of the results presented in the table. The Chao1 and ACE indices are at their peak in Group F, as illustrated in Figure 7B. Group C has the highest Shannon and Simpson indices, with Group F following closely behind. The results indicate that the bacterial population underwent considerable changes during air-drying and storage. The air-drying process may reduce the richness and uniformity of the bacterial community in sauce-flavored sausages. The cold storage phase is a crucial fermentation step in the creation of sauce-flavored sausages, significantly affecting the microbial composition of the samples.

Beta diversity was assessed by NMDS analysis. Each dot on the graph represents a sample, with dots of the same color belonging to the same subgroup. The closer the two dots are, the more similar their community compositions are. The NMDS analysis in Figure 7C shows big changes in the phylogenetic distances between the five groups when the stress value is less than 0.05, which means the graph is well represented. The analysis revealed a notable disparity in colony composition between D0 and D25, although the community makeup of the remaining sample groups (D5, D10) and D25 was comparable.

### 3.6. Composition of the Bacterial Community

**Figure 8:**
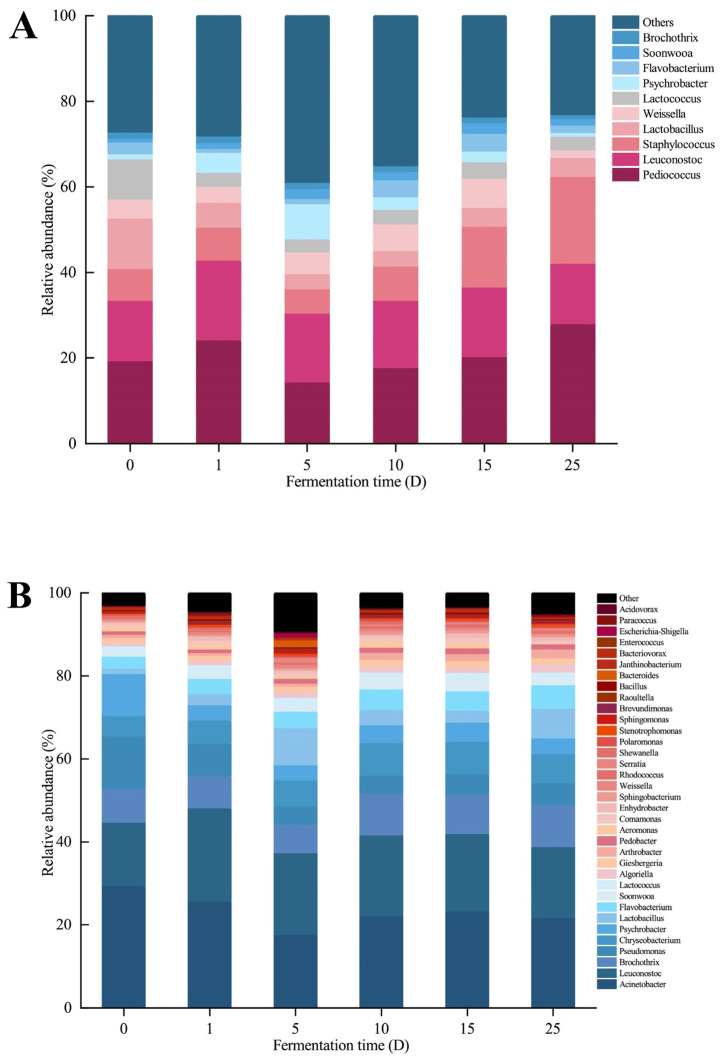
Relative abundance of bacteria at the phylum (**A**) and genus (**B**) levels of sauce-flavored sausages from 6 samples.

The study looked at bacterial 16S rRNA genes and sorted sequencing data by phylum and genus to see how the microbe community changed while the sausage was fermenting. Figure 8A displays 22 bacterial phyla identified during different fermentation stages. Among them, eight phyla had relative abundances exceeding 0.1%: *Proteobacteria*, *Firmicutes*, *Actinobacteria*, *Bacteroidetes*, *Patescibacteria*, *Verrucomicrobia*, *Chloroflexi*, and *Cyanobacteria*. *Proteobacteria* and *Firmicutes* were the two main phyla identified in Sichuan sausages, with average relative abundances of 49.78% and 30.84%, respectively. The bacteria in Sichuan sauce-flavored sausage mainly come from fermented condiments and raw meat. *Proteobacteria* and *Firmicutes* were the main bacterial flora in Pixian Doubanjiang [57,58]. These two kinds of strains were also found in the traditional dry sausages [59], sufu [60], and suancai [61]. This demonstrated that *Proteobacteria* and *Firmicutes* had played an important role in Sichuan traditional fermented foods.

At the genus level, the microbial species were spread among 392 genera. In total, 35 bacterial genera had a relative abundance greater than 0.1%, as shown in Figure 8B. The main genera found in the sausages were *Acinetobacter* and *Leuconostoc*, with 20.31% and 16.25% of the total abundance, respectively. *Acinetobacter* is the main bacterium found in meat maintained at low temperatures, and fermented sausages are typically stored at low temperatures, allowing *Acinetobacter* to proliferate and colonize [62]. *Leuconostoc* is a prevalent lactic acid bacterium found in traditional dry sausages and various other fermented foods and beverages [59,63,64]. Research indicates that strains of the bacterium *Leuconostoc carnosum* may enhance the stability and flavor of fermented meat products [65]. The proliferation of these bacteria may be the primary cause of the fall in pH. *Pseudomonas* and *Psychrobacter* declined significantly from 11.79% and 9.37% to 3.50% and 3.01% between days 1 and 5 of air-drying. Their levels remained constant over the subsequent chilling period, possibly due to the quick reduction in water activity. *Lactobacillus* levels rose quickly from 1.19% on day 1 to 7.25% on day 5, then dropped to 3.20% on day 10, and finally increased to 5.87% on day 25. *Soonwooa*, *Algoriella*, and *Arthrobacter* showed a progressive growth during fermentation, surpassing 1% and eventually becoming the predominant bacteria in the sausages.

### 3.7. Correlation Analysis

**Figure 9:**
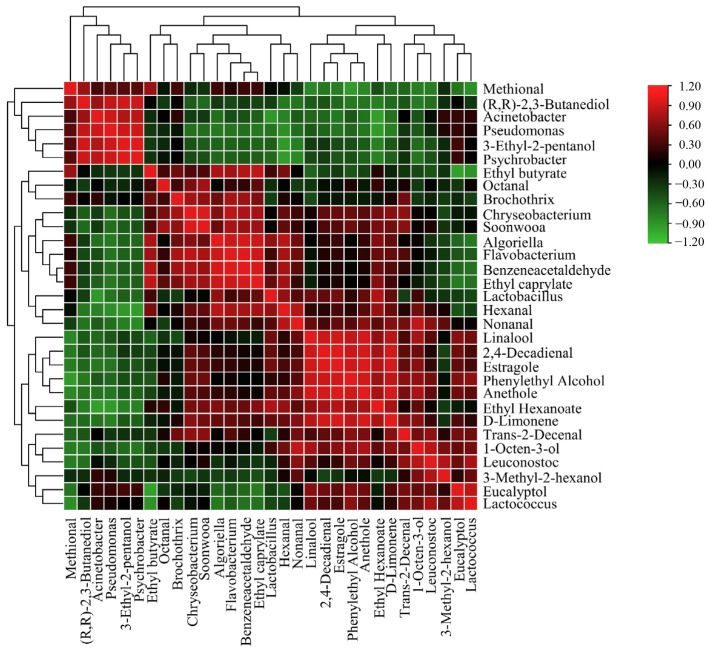
Correlation map of flavor (ROAV ≥ 1) compounds with microorganisms in the sauce-flavored sausages during fermentation. Each square indicates the Pearson’s correlation coefficient values (r). Positive (0 < r < 1) and negative (−1 < r < 0) correlations are indicated in green and red, respectively.

**Figure 10:**
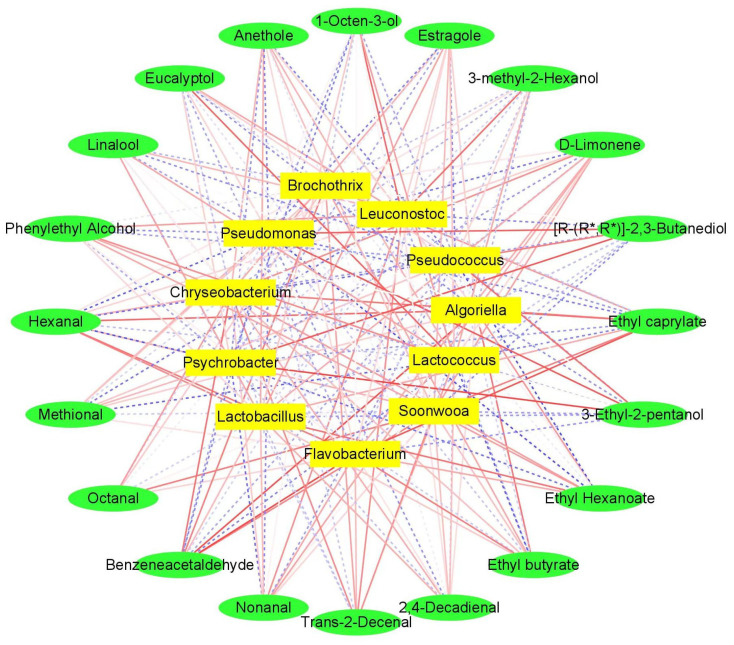
Correlative network model for the correlation between flavor compounds and microbial genera sauce-flavored sausage during fermentation.

Volatile chemicals can be influenced by raw materials, auxiliary materials, bacterial populations, and fermentation conditions [66]. The bacterial population can significantly impact the quality of sausage products and the volatile flavor compounds generated by the microorganisms’ metabolism and interactions within the bacterial communities. The core microorganisms related to sauce-flavored sausage flavor formation were identified based on three criteria: stability in the fermentation process, significant changes in relative abundance during fermentation (*p* < 0.05), and a strong correlation with volatile flavor substances (|r| > 0.8). Based on Pearson correlation coefficients, a correlation analysis was conducted between bacteria with a relative abundance greater than 1% and volatile compounds with an ROAV greater than 1 (Figure 9). Additionally, a Cytoscape network diagram was used to visually represent the relationships between the bacterial community components and key volatile compounds in sauce-flavored sausages (Figure 10). From Figure 9 and Figure 10, it can be seen that 11 different core bacterial communities (at the genus level) were observed in the samples, involving 20 key volatile flavor compounds. The results indicate that seven different genera in the samples (*Pseudomonas*, *Psychrobacter*, *Flavobacterium*, *Leuconostoc*, *Lactococcus*, *Acinetobacter*, and *Algoriella*) have significant correlations with the volatile flavor compounds.

3-ethyl-2-pentanol was positively correlated with *Pseudomonas* (|r| >0.9, *p* < 0.05) and *Psychrobacter* (|r| >0.8, *p* < 0.05), but *Psychrobacter* was primarily negatively correlated with nonanal (|r| >0.8, *p* < 0.05). *Leuconostoc* (|r| >0.8, *p* < 0.05) was found to be positively related to 1-octen-3-ol. *Algoriella* (|r| >0.8, *p* < 0.05) was primarily positively correlated with hexanal. However, in the fermentation process, this compound was primarily negatively correlated with *Pseudomonas* (|r| > 0.8, *p* < 0.05) and *Psychrobacter* (|r| > 0.9, *p* < 0.01). Bacteria can promote the degradation of proteins in meat products into free amino acids, which can be directly or indirectly metabolized into aldehydes under the action of microbial enzymes [15]. These microorganisms may also influence flavor formation by affecting lipid oxidation. Meanwhile, *Pseudomonas* (|r| >0.8, *p* < 0.05) and *Psychrobacter* (|r| >0.9, *p* <0.05) were found to be positively correlated with (R,R)-2,3-butanediol, indicating that these microorganisms could promote carbohydrate metabolism.

Eucalyptol, a common flavor enhancer, was well correlated with *Lactococcus* (|r| > 0.8, *p* < 0.05). Benzeneacetaldehyde, which is related to amino acid metabolism, correlated well with *Flavobacterium* (|r| > 0.9, *p* < 0.01) and *Algoriella* (|r| > 0.9, *p* < 0.01). Microbial esterification was also essential in the production of sausage flavor. Ethyl caprylate was positively correlated with *Flavobacterium* (|r| > 0.9, *p* < 0.05) and *Algoriella* (|r| > 0.8, *p* < 0.05). *Pseudomonas* (|r| > 0.9, *p* < 0.01) and *Acinetobacter* (|r| > 0.9, *p* < 0.01) were negatively correlated with ethyl hexanoate. *Lactococcus* (|r| > 0.8, *p* < 0.05) was primarily inversely related to ethyl butyrate. Overall, the formation of the flavor of sauce-flavored sausage products is closely related to the growth and metabolism of their microorganisms; it is not only associated with a particular microbial genus but also with the interactions of many microorganisms in the fermentation system. The most important species that promoted the formation of sauce-flavored sausage flavor substances were *Leuconostoc*, *Pseudomonas*, *Psychrobacter*, *Flavobacterium*, and *Algoriella*. Furthermore, texture and taste are important physical and chemical indicators for assessing the sensory quality of sauce-flavored sausage. We will further investigate the effect of microbial succession on these components during the fermentation of sauce-flavored sausage.

## 4. Conclusions

We have observed the bacteria community succession and dynamic changes in volatile flavor compounds during the fermentation of Sichuan sauce-flavored sausage in this study. Our findings shed light on the metabolically active bacteria that promote metabolite production during Sichuan sauce-flavored sausage fermentation. During fermentation, *Acinetobacter*, *Leuconostoc*, *Brochothrix*, *Pseudomonas*, *Chryseobacterium*, *Psychrobacter*, and other genera were dominant. Additionally, volatile compounds were detected in the sauce-flavored sausage, giving it a strong and distinct aroma. The primary flavor compounds in Sichuan sauce-flavored sausage were 1-octen-3-ol, eucalyptol, linalool, octanal, methional, 2,4-decadienal, ethyl hexanoate, ethyl caprylate, and d-limonene. These mixtures provide sausages with aromas of fruit, fat, flowers, grass, mushrooms, and more. *Leuconostoc*, *Pseudomonas*, *Psychrobacter*, *Flavobacterium*, and *Algoriella* were significantly and positively correlated with the formation of characteristic flavor substances. These findings will aid in understanding the key bacteria that affect volatile flavor compounds in Sichuan sauce-flavored sausage. They will also assist in the isolation and screening of microbial strains for the production of safe and high-quality Sichuan sauce-flavored sausage products. Further research should focus on isolating and identifying core microbiomes that are beneficial for flavor development in sauce-flavored sausages. It is important to use multi-omics, such as genomics, proteomics, lipidomics, and metabonomics, to clarify the underlying mechanisms or functions of the microbiome. Additionally, the relationship between non-volatile compounds (lipids and metabolites) and microbes of sauce-flavored sausages should be considered as a supplement.

## Figures and Tables

**Table 1:** Identification of key flavor compounds during sauce-flavored sausage fermentation.

Compounds	ROAV						Average Value of ROAV
	D0	D1	D5	D10	D15	D25	
3-ethyl-2-pentanol	6.32	0.00	0.00	1.55	0.00	0.00	1.31
(R,R)-2,3-butanediol	5.72	1.63	1.13	1.26	0.32	1.76	1.97
3-methyl-2-hexanol	0.00	10.44	0.00	0.00	0.00	0.00	1.74
1-octen-3-ol	0.00	41.10	23.22	23.50	6.22	3.57	16.28
Eucalyptol	32.04	91.25	59.16	80.42	24.66	0.00	47.95
Linalool	5.05	22.16	25.86	21.27	9.41	3.64	14.58
Phenylethyl alcohol	0.62	8.10	10.72	11.14	6.25	0.17	6.17
Hexanal	0.00	20.71	14.32	13.67	10.18	7.70	11.11
Octanal	0.00	0.00	0.00	0.00	99.24	0.00	16.67
Methional	100.00	100.00	40.61	34.09	76.34	100.00	75.27
Benzeneacetaldehyde	0.00	5.30	6.59	17.61	13.52	10.17	8.88
Nonanal	0.00	22.90	10.67	15.40	4.62	4.93	9.76
Trans-2-decenal	0.00	4.55	0.00	6.40	2.26	0.00	2.20
2,4-Decadienal	0.00	0.00	100.00	100.00	26.21	0.00	37.74
Ethyl butyrate	0.00	0.00	0.00	0.00	9.16	8.21	2.91
Ethyl hexanoate	11.95	48.00	52.93	41.64	35.88	15.50	34.36
Ethyl caprylate	11.29	52.41	71.66	87.27	100.00	56.07	63.25
D-limonene	4.43	21.15	26.46	31.84	14.41	6.28	17.45
Estragole	1.83	8.55	15.10	14.34	6.63	2.11	8.10
Anethole	1.08	5.81	6.20	6.21	3.05	1.16	3.92

**Table 2:** Number of observed operational taxonomic units (OTUs), diversity indexes (Shannon and Simpson), diversity richness (Chao 1 and ACE), and estimated sample coverage for 16S rRNA amplicon (Good’s coverage) for sauce-flavored sausage from different samples.

Sample	Observed OTUs	ACE	Chao1	Shannon	Simpson	Good’s Coverage
D0	1254.333 ± 33.546	2054.606 ± 112.144	2026.283 ± 192.269	5.490 ± 0.170	0.935 ± 0.005	0.994 ± 0.001
D1	1192.667 ± 30.667	1978.732 ± 92.051	1946.613 ± 29.750	4.690 ± 0.309	0.838 ± 0.023	0.995 ± 0.000
D5	1222.333 ± 94.044	2041.081 ± 202.660	2035.733 ± 267.214	5.933 ± 0.391	0.952 ± 0.012	0.995 ± 0.001
D10	1297.667 ± 20.744	2088.203 ± 105.973	2029.698 ± 141.262	5.758 ± 0.100	0.946 ± 0.007	0.995 ± 0.000
D15	1234.333 ± 51.509	2035.243 ± 38.418	1989.203 ± 49.049	5.594 ± 0.122	0.942 ± 0.005	0.994 ± 0.000
D25	1305.667 ± 21.385	2220.829 ± 50.905	2117.476 ± 62.372	5.770 ± 0.057	0.951 ± 0.006	0.994 ± 0.000

## Data Availability

The original contributions presented in the study are included in the article. Further inquiries can be directed to the corresponding author.
